# Spatiotemporal Modeling of the Key Migratory Events During the Initiation of Adaptive Immunity

**DOI:** 10.3389/fimmu.2019.00598

**Published:** 2019-04-05

**Authors:** Alan J. Hayes, Sanket Rane, Hannah E. Scales, Gavin R. Meehan, Robert A. Benson, Asher Maroof, Juliane Schroeder, Michio Tomura, Neil Gozzard, Andrew J. Yates, Paul Garside, James M. Brewer

**Affiliations:** ^1^Institute of Infection, Immunity and Inflammation, College of Medical, Veterinary and Life Science, University of Glasgow, Glasgow, United Kingdom; ^2^Department of Pathology and Cell Biology, Columbia University Medical Centre, New York, NY, United States; ^3^UCB Celltech, Slough, United Kingdom; ^4^Laboratory of Immunology, Faculty of Pharmacy, Osaka Ohtani University, Osaka, Japan

**Keywords:** cell migration, dendritic cells, adaptive immunity, innate immunity, cell tracking

## Abstract

Initiation of adaptive immunity involves distinct migratory cell populations coming together in a highly dynamic and spatially organized process. However, we lack a detailed spatiotemporal map of these events due to our inability to track the fate of cells between anatomically distinct locations or functionally identify cell populations as migratory. We used photo-convertible transgenic mice (Kaede) to spatiotemporally track the fate and composition of the cell populations that leave the site of priming and enter the draining lymph node to initiate immunity. We show that following skin priming, the lymph node migratory population is principally composed of cells recruited to the site of priming, with a minor contribution from tissue resident cells. In combination with the YAe/Eα system, we also show that the majority of cells presenting antigen are CD103^+^CD11b^+^ dendritic cells that were recruited to the site of priming during the inflammatory response. This population has previously only been described in relation to mucosal tissues. Comprehensive phenotypic profiling of the cells migrating from the skin to the draining lymph node by mass cytometry revealed that in addition to dendritic cells, the migratory population also included CD4^+^ and CD8^+^ T cells, B cells, and neutrophils. Taking our complex spatiotemporal data set, we then generated a model of cell migration that quantifies and describes the dynamics of arrival, departure, and residence times of cells at the site of priming and in the draining lymph node throughout the time-course of the initiation of adaptive immunity. In addition, we have identified the mean migration time of migratory dendritic cells as they travel from the site of priming to the draining lymph node. These findings represent an unprecedented, detailed and quantitative map of cell dynamics and phenotypes during immunization, identifying where, when and which cells to target for immunomodulation in autoimmunity and vaccination strategies.

## Introduction

Initiation of adaptive immunity involves a number of migratory and tissue resident cell populations coming together in a highly dynamic and spatially organized process. A detailed understanding of the spatiotemporal organization of this process is a significant goal in basic immunology. Furthermore, this information would also allow the rational development of new therapeutics and immune modulators, ensuring the correct cells are targeted at the correct location and time to optimize efficacy while minimizing off target effects. However, we currently lack a definitive description of the spatiotemporal organization of immune cell migration leading up to initiation of adaptive immunity. This is mainly due to our inability to faithfully track cells between spatially distinct locations or definitively identify these migratory cell populations. Current approaches to study cell migration include for example, transfer of exogenously labeled cells (1–3), injection of labeled dyes or analysis of cell markers associated with migration. However, these approaches suffer from issues of physiological relevance, fidelity to pharmacodynamics and distribution and a lack of functional link to cell migration as well as having a bias toward cell populations known to express that marker, respectively. An ideal cell tracking, or fate-mapping approach should therefore address these issues.

The Kaede mouse ubiquitously expresses the photo-switchable fluorescent protein Kaede that undergoes a beta elimination reaction following exposure to violet light resulting in a shift in excitation and emission (4, 5). Photoswitching is easily identified in the emission shift from green (518 nm) to red wavelengths (582 nm) using conventional flow cytometry or fluorescence microscopy. The Kaede system therefore offers the unique potential to track all of the cell populations originating from a site of light exposure within the whole-organism. This approach therefore labels cells *in situ*, addressing issues of physiological relevance associated with cell transfer and pharmacokinetic issues associated with administration of fluorescent dyes such as FITC (6, 7). Furthermore, because all of the cells in a tissue are labeled, this does not require prior knowledge of the migratory cells of interest, overcoming a biased approach to studying cell migration. Due to the advantages of Kaede mice over these methods, they provide an excellent tool to further our current understanding of cell migration.

In this study, we apply the Kaede transgenic mouse model, to produce a definitive, spatially and temporally resolved map of cell migration between the site of immunization and the draining lymph node during initiation of an adaptive immune response. We use the unbiased and functional identification of migratory cells offered by this system, to perform comprehensive phenotypic identification of cells using mass cytometry. Significantly, we show that contrary to current dogma, the majority of cells migrating from the site of immunization to the draining lymph node (dLN) are not tissue resident, however are recruited into the tissue following immunization with defined kinetics. Thus, we have defined in unprecedented detail, when, where and what cells are involved in initiation of an adaptive immune response, generating a fundamental reference source, and importantly informing the rational targeting of immunotherapy *in vivo*.

## Materials and Methods

### Mouse Strains

C57BL/6 mice were purchased from Envigo at 6 weeks of age. The Kaede transgenic mouse colony was kindly provided by Tomura (5). All animals were maintained under standard conditions at the University of Glasgow Central Research Facility and all procedures were carried out according to UK Home Office regulations.

### Photo-Conversion of Kaede Mouse Tissue

Photo-conversion of the Kaede mouse tissue was performed using a 12x S06J bluray diode with a 405-G-2-glass lens (DTR's Laser Shop) emitting at 405 nm with mean power of 600–650 mW. Post-immunization, mice were anesthetized and the ventral and dorsal side of the hind paw were illuminated with the laser for three 5 s bursts, with a 3 s interval between each burst, as previously optimized (data not shown). Photo-conversion was only performed once per animal.

### Immunization, Adjuvants, and Antigen

Mice were subcutaneously injected with 25 μl of 5 mg/ml aluminum hydroxide gel adjuvant (alum) (A kind gift from Dr. Erik Lindblad, Brenntag Biosector, Denmark) solution containing 8 μg of *E. coli* lipopolysaccharide (LPS) (serotype: 055:B5; Sigma Aldrich, UK) in the hind footpad, under isoflurane induced general anesthesia. 50 μg of an Eα peptide (I-Eα 52–68 of I-E^d^)—Ovalbumin conjugate (Eα:OVA) (ALMAC, Scotland) was also injected subcutaneously to track antigen presentation using the Y-Ae monoclonal antibody (8, 9).

### Flow Cytometry

Skin was removed from the hind paw, minced and digested in 100 U/ml of DNAse, 2 mg/ml of collagenase IV from *Clostridium hisolyticum* and 2 mg/ml of hyaluronidase from bovine testes (Sigma Aldrich). The remainder of the paw was teased apart with tweezers and added to the digestion mix. Samples were incubated for 20 min at 37°C in a shaking incubator. Following digestion, samples were passed through 100 μm cell strainers. The popliteal lymph nodes were gently passed through a nitex mesh (Cadisch Precision Meshes) using the rubber end of a 1 ml syringe plunger and digested in 2.68 mg/mL of collagenase D from *Clostridium hisolyticum* (Roche) for 25 min at 37°C in a shanking incubator. 100 μl of 100 μM EDTA was added to each sample to halt the reaction (10). Prior to staining 10 μl of each sample was removed and used to enumerate the total number of cells per sample using a hemocytometer, dead cell exclusion was performed with trypan blue. Samples were passed through a nitex mesh for a second time to generate a single cell suspension and incubated in 50 μl of Fc block [2.4G2 grown in-house (9)] containing 5% mouse serum for 10 min and subsequently stained with combinations of the following antibodies for 20–30 min: anti-CD11b (M1/70), anti-CD11c (HL3), anti-CD45 (30-7-11), anti-MHCII (M5/114.15.2), anti-Ly6G (IA-8), biotinylated YAe (eBio-YAe) (all eBioscience, Hartfield, UK), and anti-CD103 (2E7) (BD Biosciences). Detection of biotinylated YAe was performed using Streptavidin APC-eFluor®780 (eBioscience). Cell viability was measured using fixable eFluor®780 viability dye (eBioscience). Red and green Kaede were detected using the PE and FITC channels, respectively. Data was acquired on a LSRII flow cytometer running FACSDiva software (BD bioscience) and subsequently analyzed using Flowjo software (Tree star, Inc., USA). Gating strategies for flow cytometry experiments are detailed in Supplementary Figure 1.

### Mass Cytometry

Prior to mass cytometry, fixable eFluor®780 viability dye, Kaede red, and Kaede green cells were sorted on an FACSAriaII cell sorter running FACSDiva software. Sorted cells were collected in 50% FCS. For mass cytometry, samples were incubated with the following antibodies for 2 h at 4°C: anti-CD3e (145-2C11), anti-CD4 (RM4-5), anti-CD8α (53-6.7), anti-CD11b (M1/70), anti-CD11c (N418), anti-CD19 (6D5), anti-CD25 (3C7), anti-CD44 (IM7), anti-CD45 (30-F11), anti-CD62L (MEL-14), anti-CD69 (H1.2F3), anti-CD86 (GL1), anti-CD103 (2E7) anti-CD127 (A7R34), anti-CD197 (4B12), anti-APC (APC003), anti-B220 (RA3-6B2), anti-biotin (1D4-C5), anti-CTLA-4 (UV10-4B9), anti-F4/80 (BM8), anti-Ly6G (RB6-8C5), anti-MHCII (M5/114.15.2), anti-NK1.1 (PK136), anti-TcRβ (H57-597), and YAe (eBioY-Ae) (Fluidigm, South San Francisco). Cells were then stained with DNA intercalator overnight at 4°C before being re-suspended in 500 μl of ultrapure water for data acquisition on a CyTOF 2. Analysis was performed using Flowjo software (Tree star, Inc.) and Cytobank software (Cytobank, Inc.). The gating strategy used for mass cytometry is detailed in Supplementary Figure 2.

### Mathematical Modeling

We used an array of mathematical models to describe the dynamics of DC migrating between a site of inflammation and the dLN. We fitted each model simultaneously to the time courses of cell numbers in the two sites, under the different photoswitching strategies. This process characterized (i) the dynamics of egress of DC from skin over the 72 h following challenge, (ii) transit between skin and the dLN, and (iii) the distribution of residence times of migratory DC within the dLN. The models are described in detail in Supporting Information 1. Model simulation and parameter estimation was performed in *R*, using nls() and optim(), the latter employing the Nelder-Mead algorithm. Model selection was performed using the Akaike information criterion (AIC).

### Statistical Analysis

Results are presented as individual values with the mean, or the mean individually ± 1 standard deviation (SD). Groups were compared using a one-way ANOVA or a two-way ANOVA. All statistical analysis was performed using Prism software (Graph pad software, Inc.). *P* ≤ 0.05 were considered to be statistically significant.

## Results

### Photo-Conversion of the Kaede Protein Does Not Impact Cell Viability or Activation Status

In order to assess the effect of the 405 nm laser diode on the kaede protein and cell viability we exposed lymph nodes from kaede mice to the laser for 2–30 s *ex-vivo* and assessed kaede fluorescence and viability by flow cytometry. The 405 nm laser diode could successfully induce photo-conversion of Kaede lymph node cells within 2 s of exposure (Supplementary Figures 3A,B without loss of cell viability (Supplementary Figure 3B). In addition, photo-conversion using the 405 nm laser diode did not induce changes in the activation status of bone marrow-derived dendritic cells (BMDCs) *in vitro* as assessed by expression of MHCII, CD80, and CD86 (Supplementary Figures 3C–E), nor did exposure alter the ability of BMDCs to upregulate activation markers CD80, CD86, and CD40 in response to LPS stimulation (Supplementary Figures 3C–E).

### Lymph Node Migratory Cell Population Is Almost Completely Comprised of Inflammatory, Rather Than Skin Resident Cells

**Figure 1:**
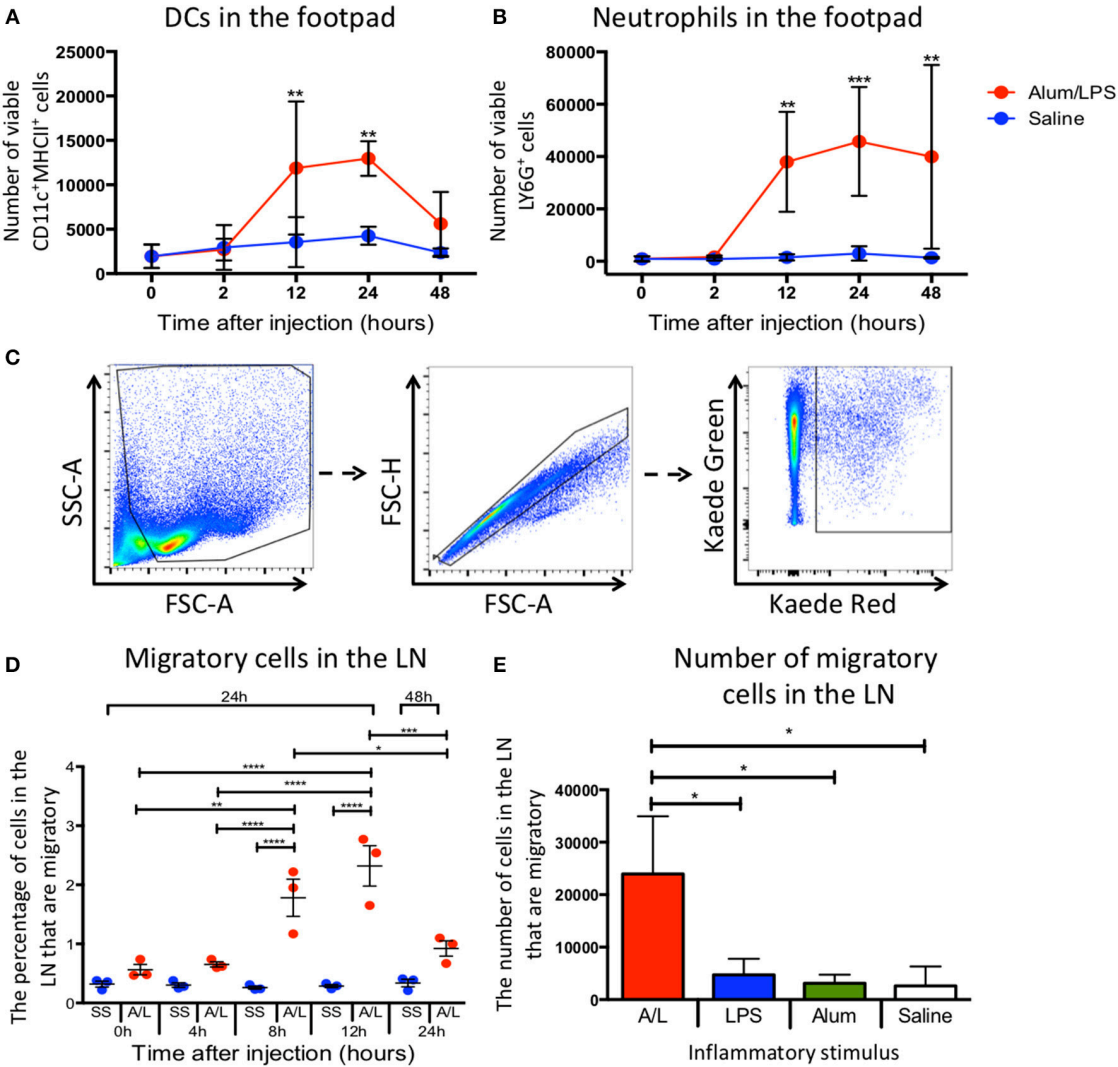
Inflammation at the site of injection drives migration to the draining lymph node. C57BL/6 mice were injected with alum/LPS in the left hind footpad and saline in the right hind footpad and subsequently culled 0, 2, 12, 24, or 48 h later and the skin from the footpads analyzed by flow cytometry. **(A)** The total number of live CD11c^+^MHCII^+^ cells isolated from the footpad and **(B)** the total number of live Ly6G^+^MHCII^−^ cells isolated from the footpad are shown. *n* = 4 ± 1 SD (^**^p < 0.01, ^***^p < 0.001). Kaede mice were injected with alum/LPS in the left hind footpad or saline in the right and the footpad was photo-converted at 0, 4, 8 or 12 h post injections. Mice were then culled 24 or 48 h after injection and the popliteal lymph node removed and analyzed by flow cytometry. **(C)** Migratory cells were identified by gating out debris using FSC and SSC, doublet exclusion using FSC-A and FSC-H followed by identifying migratory cells based on Kaede red expression. As we exposed the footpad to violet light only, any cells expressing Kaede red in the lymph node must have migrated there from the footpad. **(D)** The percentage of migratory cells entering the draining lymph node directly from the footpad is shown. *n* = 3 ±1 SD (^*^p < 0.05, ^**^p < 0.01, ^***^p < 0.001, ^****^p < 0.0001). **(E)** the number of inflammatory cells identified in the draining lymph node of Kaede mice after challenge with alum alone, LPS alone or alum/LPS over a 24 h period is shown. *n* = 3 ± 1 SD (^*^p < 0.05).

To assess how alum/LPS altered cellular composition at the site of injection, we challenged mice in the hind footpad with alum/LPS or saline and assessed the number of DCs and neutrophils at the injection site by flow cytometry. Injection with alum/LPS produced no significant change in the number of DCs (CD11c^+^MHCII^+^) or neutrophils (CD11b^+^Ly6G^+^) at the injection site compared with saline treated animals after 2 h (Figures 1A,B). However, alum/LPS induced a significant increase in both DC and neutrophil numbers 12 h after injection which remained elevated at 24 h. 48 h post-injection the number of DCs began to reduce, while the number of neutrophils remained significantly increased. The inflammatory site therefore creates a dynamic environment with changing cell numbers and profiles, that could impact on the phenotype of the LN migratory population.

To successfully track cell migration from the footpad to the draining lymph node we used the kaede transgenic mouse. The kaede mouse ubiquitously expresses the Kaede protein in all cells. When the protein is exposed to violet light, there is a change in fluorescence from green to red. By inducing photo-conversion locally in the footpad, we can track the cells present at the time of photo-conversion as they migrate to more distal sites such as the dLN. This system allows us to fate map migratory cells without relying on differentially expressed markers such as CD103, the expression of which can be dynamically regulated during an immune response (11). Therefore, we challenged Kaede mice with alum/LPS or saline in the hind footpad, and photo-converted the injection site 0, 4, 8, 12, or 24 h post-injection. Any cells expressing red Kaede in the dLN must have originated at the site of photo-conversion and will henceforth be referred to as migratory cells The parameters used to identify migratory cells are highlighted (Figure 1C). Delaying photo-conversion of the footpad until 8 or 12 h post-challenge significantly increases the percentage of migratory cells that can be identified in the dLN (Figure 1D). This suggests that the majority of the LN migratory cells during the inflammatory response to alum/LPS are not tissue resident cells and must instead be recruited to the site of challenge. We have also shown that upon stimulation with alum alone, LPS alone or saline, the number of migratory cells in the dLN does not significantly change 24 h after injection. However, when we induce inflammation using alum/LPS there is a significant increase in migratory cell number within the dLN (Figure 1E). Each inflammatory challenge is known to induce a large inflammatory response at the site of challenge, however only the combination of alum/LPS induced a significant increase in cell migration to the draining lymph node. Furthermore, alum/LPS has a synergistic effect over the individual components administered alone as the level of migration observed with a combination approach is 4 times greater than either individual treatment.

### The Migratory Population Comprises Multiple Populations, Including Antigen Presenting CD103^+^CD11b^+^ DC

**Figure 2:**
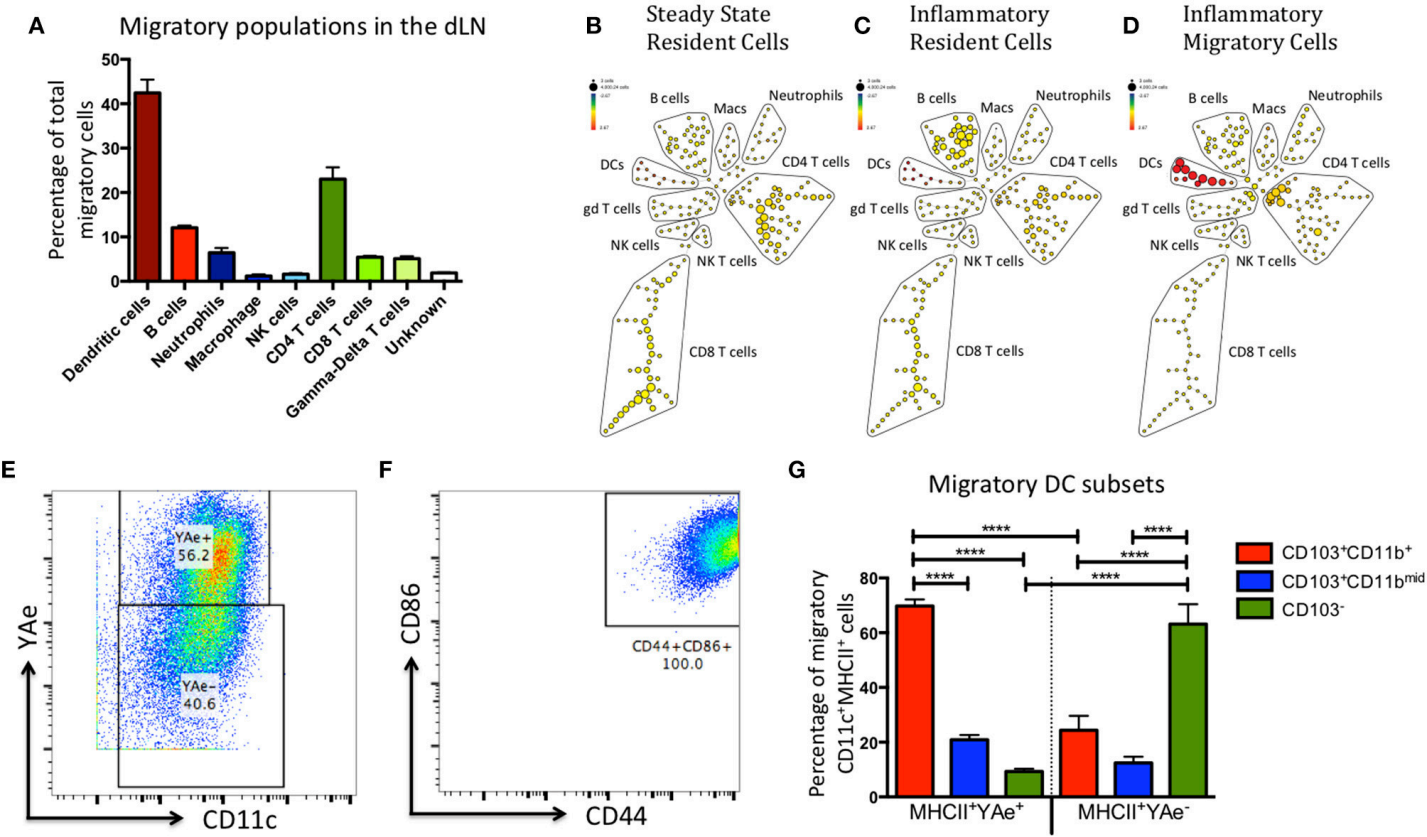
The migratory population contains diverse cell types, but the antigen presenting cells within this population are predominantly CD103^+^ CD11b^+^ dendritic cells. Kaede mice were injected with Alum/LPS/Eα-OVA in the hind footpad, 12 h later the tissue was photo-converted and the animals were culled after a further 12 h. **(A)** Analysis of the sorted, migratory population is shown with cells expressing MHCII represented in red, innate cells in blue and T cells in green. **(B–D)** The spade diagrams show the levels of antigen presentation (YAe) detected on the lymph node resident population in untreated mice, mice treated with alum/LPS in the foot pad and the migratory population of mice treated with alum/LPS in the footpad. Red and yellow nodes represent high and low expression of Eα:MHCII complexes, respectively. **(E)** Dendritic cells were identified based on expression of CD11c and MHC class II. Representative dot plots identifying antigen presenting DCs and **(F)** expression of CD44 and CD86 are shown. **(G)** The phenotype of DCs that expressed Eα:MHCII complexes and those that did not were further investigated based on the expression of CD103 and CD11b. *n* = 3 ± 1 SD (^****^p < 0.0001).

DCs are not the only cell type to migrate to the dLN upon inflammatory challenge (12–14). Therefore, to maximize the number of cell lineages we could identify and reduce potential bias from our data, we sorted the migratory population (red Kaede) and subsequently analyzed it using multi-parameter mass cytometry. Kaede mice were treated with alum/LPS containing 50 μg of Eα-OVA (alum/LPS/Eα) in the hind footpads. The tissue was photo-converted 12 h later, and the mice culled after a further 12 h. Of the migratory population, 45% expressed CD11c and MHCII (DCs), 25% expressed CD3, TcRβ and CD4 (CD4 T cells), 5% expressed CD3, TcRβ and CD8 (CD8 T cells), and a small population of cells expressed CD3 but not TcRβ (γδ T cells) (Figure 2). Another 5% of cells expressed CD11b and Ly6G (neutrophils) and 10% express MHCII, CD19 and B220 (B cells). Finally, we identified a population of cells expressing F4/80 (macrophages) and another expressing NK1.1 (NK cells) (Figure 2A). Making use of the Eα/Y-Ae system we could also show that only the migratory DCs had detectable levels of antigen presentation (Figures 2B–D). The DCs identified as migratory all had an activated phenotype (characterized by high expression of CD44 and CD86) (Figures 2E,F). The markers CD103 and CD11b are commonly used to define DC subsets in the skin, gut and LN of mice (15–19). Interestingly, the majority of DCs presenting Eα peptide on MHCII were CD103^+^CD11b^+^ and while there was a migratory population that were CD103^−^, these DCs did not present antigen (Figure 2G).

**Figure 3:**
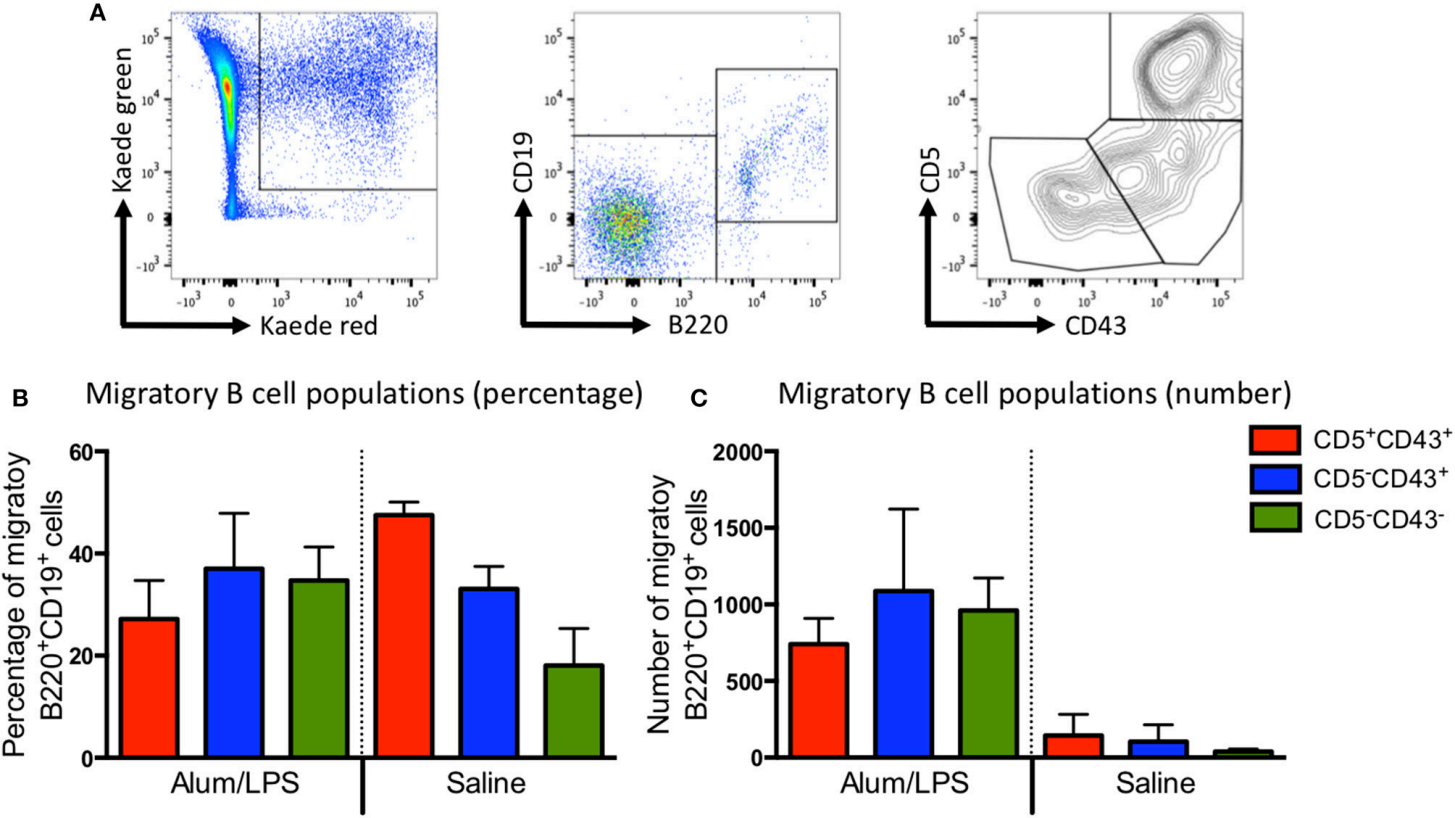
B1a, B1b, and B2 cells can be identified in the migratory population. Kaede mice were injected with alum/LPS or saline in the hind footpad and photo-conversion was performed 12 h later. Mice were culled after a further 12 h and the popliteal lymph nodes analyzed by flow cytometry. **(A)** The gating strategy used to identify B cells from the migratory population is highlight. Migratory cells were first identified and B cells selected based on B220 and CD19 expression. B cells subsets were identified using CD5 and CD43. We show **(B)** The proportion and **(C)** numbers of each B cell subset. Data are representative of two independent experiments. *n* = 3 ±1 SD.

Most of the migratory CD4 T cells expressed high levels of CD44 and low levels of CD62L, indicative of an activated phenotype (Supplementary Figure 4). Recent reports would suggest that these are likely to be T effector memory or T regulatory cells (14). The migratory B cell population could be divided into the 3 main B cell subsets (B1a, B1b, and B2) based on the expression of CD43 and CD5 (Figure 3A), all of these were present within the migratory population (Figures 3B,C). These data suggest that the migratory population is diverse with many different cell types playing a role in the control of the adaptive immune response. However, CD103^+^CD11b^+^ DCs constituted the main antigen presenting population.

### Migratory Cells Enter the Lymph Node Within the First 24 h and Migration From the Inflammatory Site Persists for 48 h After Injection

**Figure 4:**
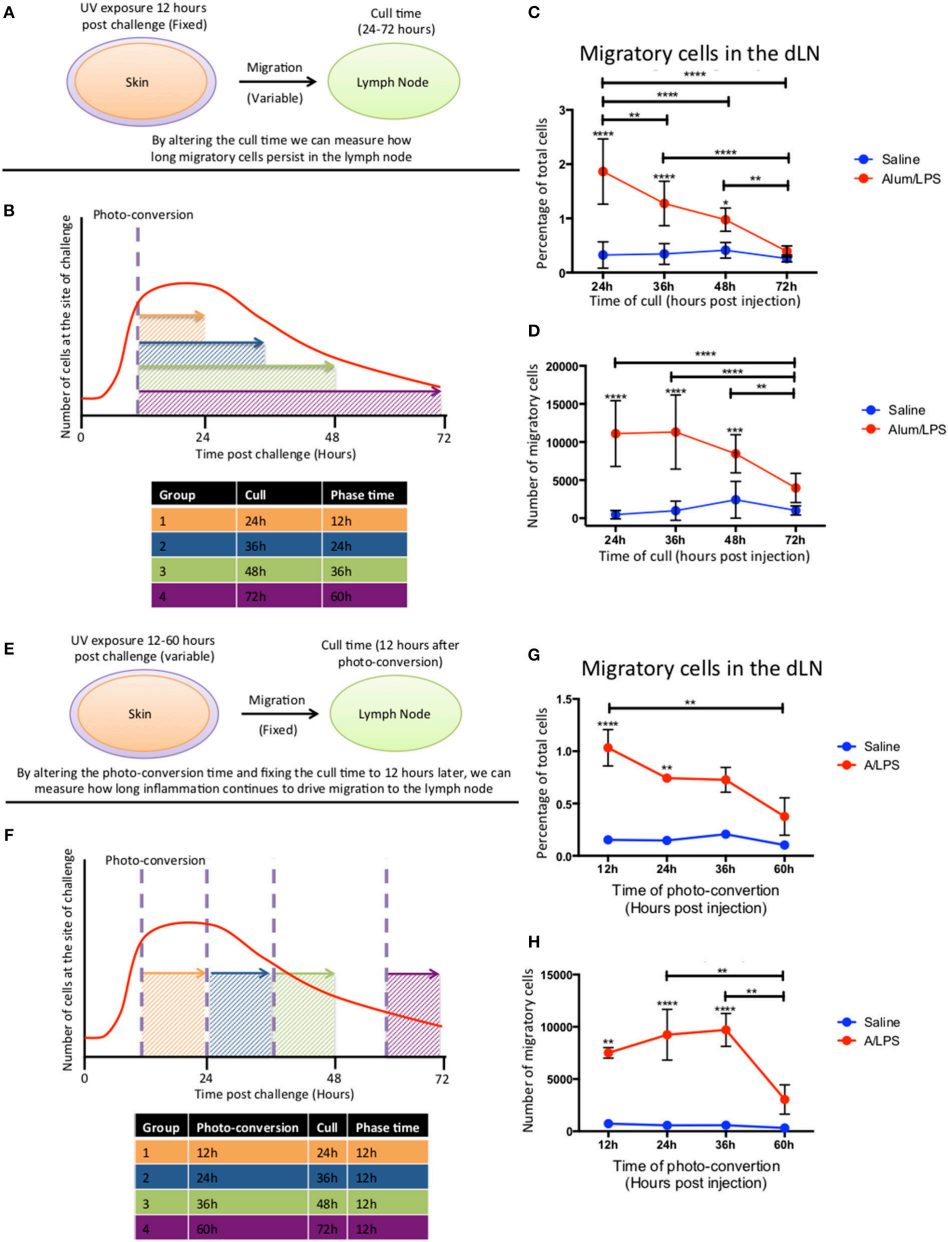
Cell migration from the site of challenge to the draining lymph node persists for 48 h post injection. **(A,B)** Footpads of mice were exposed to violet light 12 h after injection and were culled 24, 36, 48 or 72 h post injection. This strategy allowed us to quantify how long migratory cells took to travel to the dLN and how long they persisted there. **(C)** The percentage and **(D)** the total number of live migratory cells in the draining lymph node are shown. **(E,F)** Alternatively, to define how long migratory cells continue to leave the injection site, footpads were exposed to violet light at 12, 24, 36 of 60 h after injection and were culled 12 h after the photo-conversion time. **(G)** The percentage and **(H)** the total number of live migratory cells in the draining lymph node are shown. *n* = 4 ± 1 SD (^****^p < 0.0001, ^***^p < 0.001, ^**^ *p* < 0.01 and ^*^p < 0.05; data are representative of two independent experiments).

To investigate how long migratory cells persisted in the dLN, Kaede mice were challenged with alum/LPS in the hind footpad. The tissue was photo-converted 12 h later and the mice culled 24–72 h post-challenge to investigate time of arrival from the skin and persistance in the dLN (Figures 4A,B). Migratory cells could be detected in the lymph node 24 h after challenge with the percentage of migratory cells decreasing significantly over time and returning to the control levels by 72 h post injection (Figure 4C). However, the number of migratory cells remains constant between 24 and 36 h suggesting continuing migration of cells that entered the inflammatory site after 12 h and were therefore not photo-converted (Figure 4D).

To address the question of how long the inflammatory site supplies cells to the dLN, mice were injected with alum/LPS and photo-converted 12, 24, 36, or 60 h post injections and culled 12 h after photo-conversion (Figures 4E,F). The percentage of migratory cells in the dLN was significantly increased when mice were photo-converted 12 and 24 h post-injection with a subsequent decrease when photo-conversion was performed 60 h post challenge. The number of migratory cells was also significantly increased when photo-conversion was performed at 12, 24, and 36 h post-injection with a sharp decrease in migratory cell number when photo-conversion was performed at 60 h post-injection (Figures 4G,H). Based on these data we suggest that cells are continually being recruited to the site of injection before subsequently migrating to the dLN during the first 48 h following injection.

### Migratory Cells Present Antigen for 36 h Post Challenge

**Figure 5:**
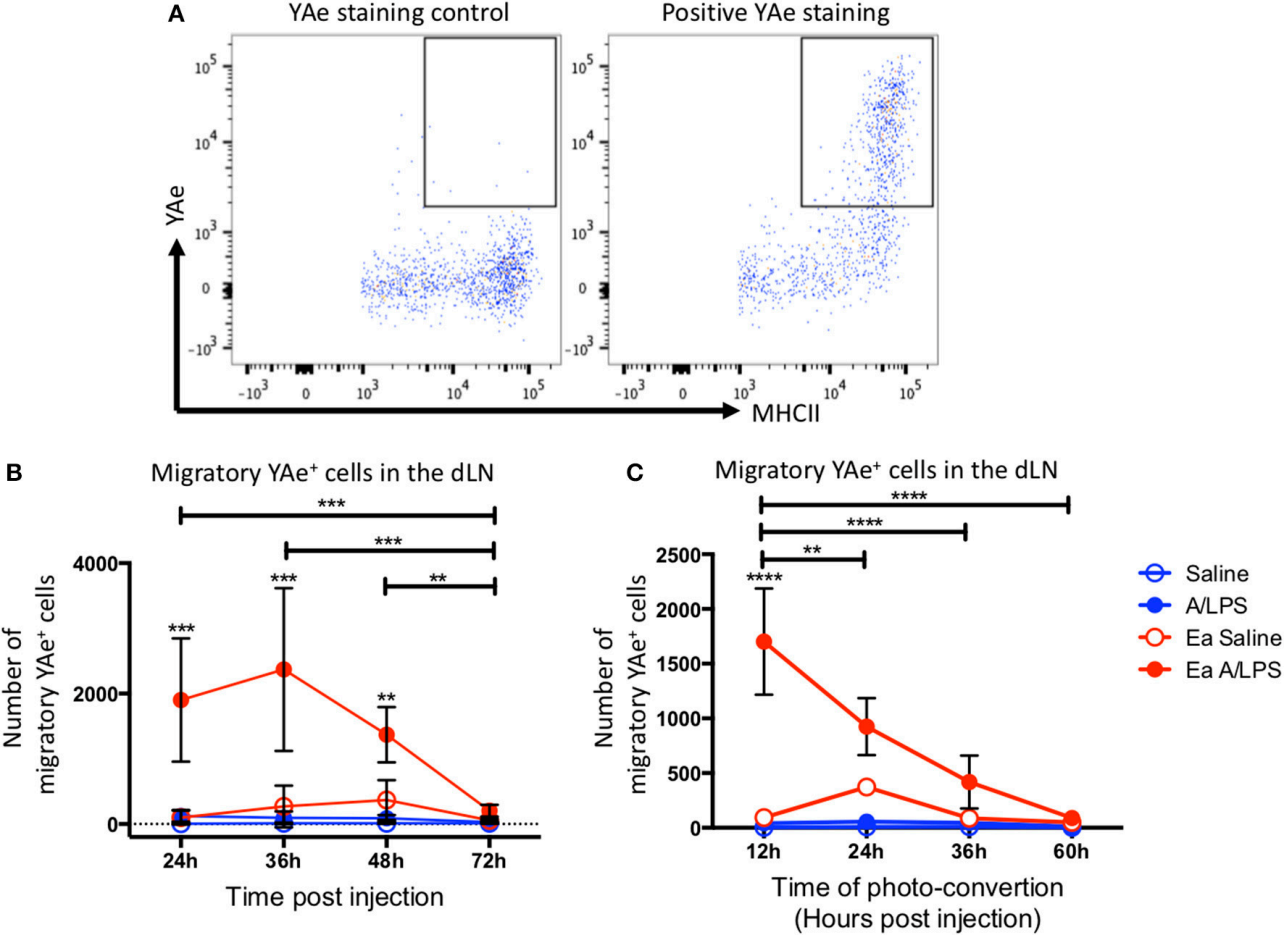
The peak of antigen presentation occurs at 24–36 h post challenge. Mice were treated with alum/LPS/Eα or saline/Eα, photoswitched 12 h later then flow cytometry analysis of isolated LN was performed between 24 and 72 h post challenge. **(A)** The identification of YAe^+^ cells was performed using a YAe staining control using the YAe antibody in samples without the Eα peptide. Positive staining in shown for comparison. **(B)** The number of live migratory MHCII^+^ cells that present antigen (YAe^+^). Data are representative of two independent experiments. *n* = 4 ± 1 SD (^**^p < 0.01, ^***^p < 0.001). Mice were treated with alum and LPS or saline with or without the Eα-OVA construct and culled between 24 and 72 h post challenge with photo-conversion performed 12 h before the cull. **(C)** The number of live migratory MHCII^+^ cells that are presenting antigen (YAe^+^) is shown. *n* = 4 ±1 SD (^**^p < 0.01, ^****^p < 0.0001).

To investigate antigen presentation within the migratory population, we combined the Y-Ae/Eα system with Kaede mice. Representative flow cytometry plots of YAe staining control and positive YAe staining highlight the effectiveness of the antibody used to identify cells presenting antigen in the dLN (Figure 5A). Photo-conversion 12 h after immunization revealed a significant increase in the number of migratory cells presenting antigen in the draining LN at 24, 36, and 48 h post-injection when compared with control animals (Figure 5B). When we varied the time of photo-conversion after immunization, but culled the mice 12 h later, we observed a significant increase in cells presenting antigen in the dLN when compared with controls when mice were photo-converted at 12 h post-challenge, with the number of cells decreasing toward baseline thereafter (Figure 5C). This kinetic data suggests that following injection with alum/LPS, the majority of antigen presentation occurs on cells that arrive at the site of injection during the first 36 h post-challenge and then take at most, 12 h to migrate to the draining lymph node.

### Modeling the Key Parameters Defining Cell Migration to the Lymph Node

**Figure 6:**
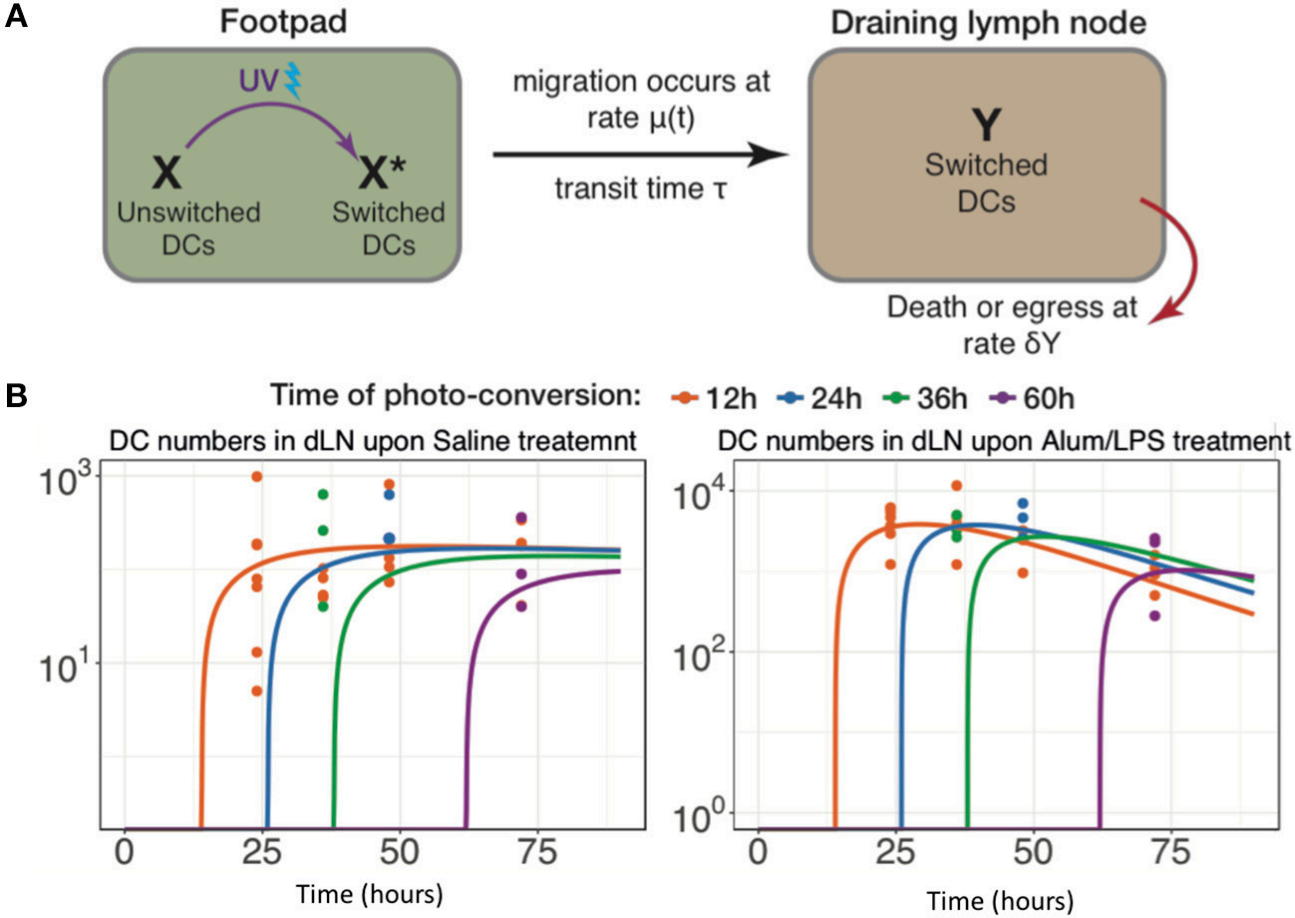
Modeling the trafficking of DC between skin and draining lymph nodes. **(A)** Schematic of the mathematical model of dendritic cell migration. Dendritic cells in the foot pad *(X)* were photo-converted (X^*^) and migrate to the lymph node at the rate μ, taking a fixed transit time τ. “*Y”* is the number of photo-converted DCs that accumulate in the dLN over time and are lost, either by death or egress, with the rate δ_*Y*_. **(B)** Fits of the model to data, showing the observed and best-fit numbers of photo-converted DCs in draining lymph nodes for saline treatment (left-hand panel) and for alum/LPS treatment (right-hand panel).

The data above highlight the limitations of describing quantitative, dynamic data using descriptive terminology, therefore we developed an array of simple mathematical models to explain DC dynamics under steady state and inflammatory conditions. We fitted the counts of photo-converted dendritic cells to a set of models considering different assumptions for the rates of migration and resident times in the draining lymph nodes in alum/LPS treated and control animals (Figure 6A). We found that the model describing constant rate of migration under steady state conditions and time-dependent changes in migration rate upon alum/LPS administration, was statistically most favored (Figure 6B).

**Figure 7:**
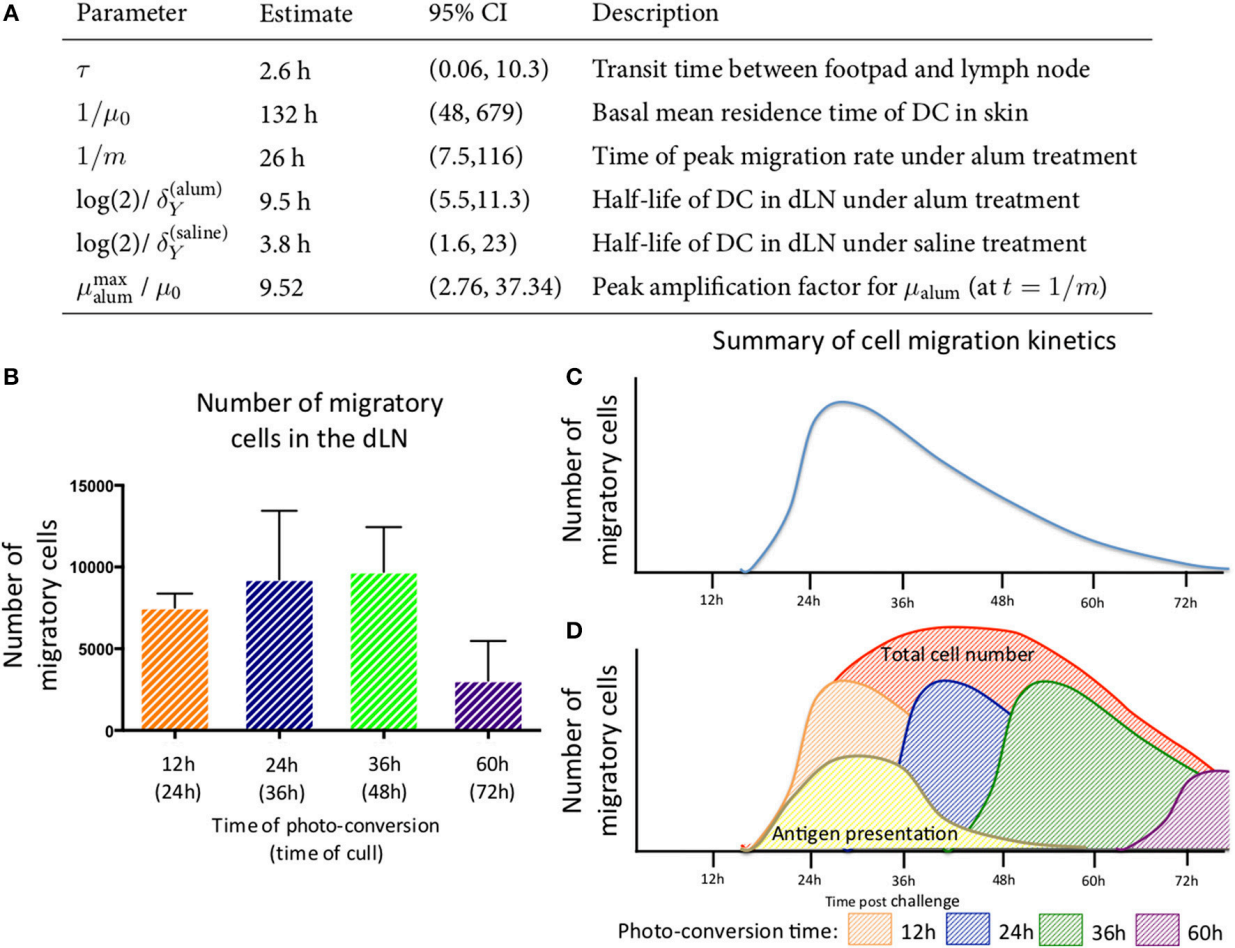
Parameter estimates for the best fitting model in which μ_alum_ = μ_0_ + αte^−mt^ and μ_saline_ = μ_0_. **(A)** the parameter estimates calculated from the mathematical model are shown. **(B)** The number of migratory cells identified in the draining lymph node at various times with **(C)** a schematic of the migration pattern observed over a 72 h period. **(D)** By combining the data generated from the experimental and mathematic approaches, it is predicted that the cells entering the tissue will follow the same rate of decay and the estimated kinetics of cell migration from the skin to the lymph node and antigen presentation are summarized.

The best-fit model depicted in Figure 6B also shows that DCs leaving an inflammatory environment survive longer and/or egress the dLN more slowly with t_1/2_ of 9.5 h in alum/LPS treated mice and t_1/2_ of 3.8 h in control groups. Our simple model considers that the transit times of DCs from the footpad to popliteal lymph nodes is not affected by the inflammatory cues in the footpad and was estimated to be 2.6 h for both treated and control animals. Lastly, we found that the migration rate of DCs in response to inflammatory conditions follows non-linear dynamics, peaking around 26 h post alum/LPS administration and is ~10-fold higher than the basal migration rate at its maximum value, implying that inflammation drives stronger and sustained migration of DCs from skin tissue to lymph nodes (Figure 7A). The mathematical model and experimental data are summarized in Figures 7B–D highlighting the peak of cell migration and antigen presentation in response to inflammatory challenge.

## Discussion

Cell migration plays a critical role in initiating and controlling the adaptive immune response (20–22). In this study, we have used the Kaede mouse system to identify the cells that leave a site of immunization in the skin and then migrate to dLN. In contrast to previous studies that suggest the migratory population is dermal resident (23–25), we have identified that the cells recruited to the site of challenge constitute the majority of the migratory population. The precise nature of Kaede and the spatiotemporal control we can exert when optically highlighting cells using this method allows for a more in-depth analysis of migratory behavior. This has allowed us to further study the origin of migratory cells which the aforementioned studies were unable to do. This likely explains why we have been able to identify migratory cells as recruited rather than tissue resident.

Having measured the kinetics of entry and egress of cells in the skin, we investigated the phenotype of the migratory population. Previous studies have identified that the skin is home to multiple DC subsets. These populations are often split based on their expression of CD103, CD11b, and CD207 (26, 27). However, CD103 and CD11b double positive DCs have not previously been identified in the skin, with the currently described subsets expressing one marker or the other. Double positive DC were previously thought only to reside in the intestine and associated mesenteric LNs. As such we report the finding of migratory CD103^+^CD11b^+^ DCs in the skin dLN as novel. In addition to being a novel finding, we also make the intriguing observation that this DC population constitutes the majority of the migratory, antigen presenting DCs in the dLN. However, it is unclear if this population of DCs has a preferential ability to acquire, process and present antigen, or if CD103 is upregulated in response to antigen acquisition or the environment of the lymph node, for example through communication with antigen-specific T cells in the lymph node in a manner similar to DC licensing (28).

In addition to DC subsets, we also identified multiple other cell types that migrate, including T and B cells. While we have not described these populations here, if they are undergoing migration in response to an inflammatory stimulus, they could participate in the initiation or regulation of adaptive immunity in the dLN. The migration of B1 B cells is of particular note, as while these cells have been described in relation to the skin (29, 30), there have been no reports of their migration to the dLN. We also identified that within the first 12 h post injection there is an increase in the number of DCs and neutrophils at the injection site in response to alum/LPS stimulation, with DC numbers increasing 3-fold and neutrophil numbers increasing 20-fold. This increase is consistent with previous reports citing an increase in neutrophils, DCs and monocytes after injection of alum or MF59 (31).

By fitting and comparing a variety of mathematical models to the data, we were able to generate a unified, quantitative view of DC dynamics in both steady state and following inflammatory stimuli. Turnover (loss) of DC in lymphoid and non-lymphoid tissues has been studied by measuring the incorporation of labels such as BrdU into the DNA of dividing cells (32–34). However, the interpretation of labeling kinetics is heavily dependent on assumptions regarding the relative contributions of influx and local proliferation, cell-cell variation in residence times, and to what extent any cells flowing into the tissue or LN are labeled (35). (To illustrate, Ruedl et al. estimated LN residence time as the time to plateau in BrdU labeling (32). If all labeled DC in the LN derive from influx, and cells leave the LN or die with first-order kinetics, as has been observed for T cells (36) it can be shown that the time to achieve a proportion *f* of LN cells labeled is log(1/f)*T* where *T* is the mean residence time. So, for example, 95% labeling is only reached after approximately 3*T*). Estimates of residence times in these studies were therefore approximate.

Relatively little is known regarding the turnover of DC in skin. Ruedl et al. saw a slow accumulation of BrdU-labeled DC in skin at steady state. From their BrdU kinetics (32), we estimate that the mean residence time of DC in skin is at least 30d if all BrdU labeled cells come from immigrants, and longer if there is any *in situ* proliferation. In our model we assume no proliferation within the skin (37), and that all egressing cells appear later in the dLN. This model yields an estimated mean skin residence time of 5.5d, though with some uncertainty (95% CI: 2–28d), which is shorter than the rough estimate obtained from Ruedl et al. but consistent at its upper bound (32).

Ruedl et al. found similarly slow accumulation of BrdU-labeled skin-derived DC in the draining LN, from which they inferred that residence times in dLN were long (32). However, Kamath et al. pointed out that the similarity of the kinetics of BrdU-labeled DC in skin and the dLN imply that turnover of DC in the LN is relatively fast and that their numbers closely reflect the flow of immigrants (33). We confirm the latter interpretation by directly estimating LN residence times of only a few hours at steady state. We do not distinguish egress and death within the LN; however previous studies using the Kaede system found very few labeled cells in other LN, supporting the conclusion that the short residence time of DC in LN is due to cell death and not egress (38).

Previous studies have reported that skin-derived DCs are first detected 3–8 h after egress from skin (38–42). However, these assays will tend to overestimate transit times because they neglect residual residence times in skin, and in most cases detection of signal is possible only after accumulation of substantial number of DCs in the dLN. Using a dynamical model accounts for these factors, and from it we estimate that DCs from skin spend an average 2.6 h (0.06, 10.3) in the lymphatics before reaching the dLN.

Various inflammatory stimuli have shown to transiently enhance the migration of skin-derived DCs to the dLNs, with a consensus that numbers of skin-derived DC reach a plateau by approximately 48 h and return to baseline level after approximately a week (38, 40, 43). We observed a similar kinetic of accumulation of DC in the dLNs following administration of Alum/LPS and show that this kinetic is driven by a transient increase in recruitment to skin and a rate of migration from skin to dLN which peaks around a day post-challenge at approximately 10-fold higher than the basal migration rate. This increase is remarkably consistent with an estimate from a previous study which used a semi-empirical approach to quantify trafficking of fluorescently labeled skin-derived DCs to dLNs, under inflammatory settings (38). Finally, our analysis shows that the skin-derived DCs persist longer in dLNs upon Alum/LPS administration as compared to control groups. This observation opens up new avenues for improving the effectiveness of DC-based vaccinations, by optimizing adjuvant concentrations for sustained migration and longer retention of DCs in dLNs.

In summary, using a combination of experiments and dynamic modeling we have mapped and quantified the processes underlying the kinetics of DC accumulation and loss in the dLN in the first few days of an inflammatory response. We find that, during the inflammatory response against alum/LPS, the majority of the DC trafficking to the dLN are newly recruited to the skin as a result of an inflammatory challenge. Alum/LPS is a homolog of the alum based vaccine AS04, and thus understanding where and when to target cells for this vaccine strategy could prove fundamental in improving It's use. The migratory population comprises diverse subsets of cells, and we show for the first time that these include a population of antigen-presenting CD103^+^CD11b^+^ DCs and B1 B cells. Our analyses can guide future efforts to ensure that the correct cells are targeted at the correct time and location to maximize efficiency and efficacy when designing therapeutics and vaccines.

## Ethics Statement

This study was carried out at the University of Glasgow and procedures were performed in accordance with UK Home Office regulations. The protocol was approved by the University of Glasgow Ethics Committee.

## Author Contributions

The experiments were conceived and designed by AH, HS, RB, NG, PG, and JB. The experiments were performed by AH, HS, GM, RB, AM, and JS. The data was analyzed by AH and HS. Provision of essential reagents and animals by MT. The mathematical modeling was carried out by SR and AY. The paper was written by AH, SR, AY, and JB. All authors have read and approved the manuscript.

### Conflict of Interest Statement

The authors declare that this study received funding from UCB Celltech, Slough. The following employees were involved in the study design and the collection and analysis of all samples analysed by mass cytometry: NG and AM. The remaining authors declare that the research was conducted in the absence of any commercial or financial relationships that could be construed as a potential conflict of interest.
