# Supplementary material for: Spatiotemporal Modeling of the Key Migratory Events During the Initiation of Adaptive Immunity
Source: Front Immunol. 2019 Apr 5;10:598. doi: 10.3389/fimmu.2019.00598 (PMC6460458; doi:10.3389/fimmu.2019.00598)
Supplement: Supplementary file 1 [file Data_Sheet_1.docx]

**Supplementary figure 1: Flow cytometry gating** The gating strategy used to define cells by flow cytometry is shown. Migratory cells were defined as shown by path A and subsequently 3 different approaches were used to define (b) dendritic cells, (c) B cells and Neutrophils and (d) T cells.

**Supplementary figure 2: Mass cytometry gating.** The gating strategy used to define population during analysis of mass cytometry data is shown. Box 1, 2 and 3 each define the stagy used to further analyse populations 1, 2 and 3 as defined by MHCII and TcRβ.

**Supplementary figure 3: Exposure to UV light does not affect cell viability or induce changes in dendritic cell activation**. Single cell suspensions isolated from lymph node were exposed to UV light for varying times and the (a) percentage of cells expressing red and green kaede and (b) the viability was measured. Bone marrow derived dendritic cells were exposed to UV light for varying times followed by a media change or LPS stimulation. Expression of (c) CD40, (d) CD80 and (e) CD86 was measured. n=3 ±1 SD (* p<0.05, ** p<0.01, *** p<0.001, **** p<0.0001)

**Supplementary figure 4: Migratory T cells can be divided into multiple populations.** Isolated Migratory cells were analysed by mass cytometry. T cells were identified based on TCR and CD3 expression. (a) CD4 and (b) CD8 t cells were further analysed based on their expression of CD44 and CD62L.

All the models followed a generalised set of assumptions listed below:

1. DCs exit the skin at *per capita* rate *μ*, such that in any short time interval *δt* each DC leaves with probability *μδt*, irrespective of their residence time in the skin. The total rate at which DCs leave the skin (cells per unit time) is therefore *μX*, where *X* is the number of DCs in the skin.
2. Considering the short time-frame of this experiment, we assume that egress is the only fate for photo-converted DCs (‘no death’). This assumption allows us to describe the change in numbers of photo-converted cells in the footpad with a simple exponential decay (Eq. 1), such that their average resident time in the footpad is 1/*μ*. We consider that the process of photo-conversion has no effect on the egress rate *μ*.
3. Every DC that leaves the skin appears in the dLN after time *τ*.
4. DC that drain to the dLN leave or die at a combined per capita rate *δY* . That is, mean residence time of DCs in dLN is *1/δY* and half-life is *ln(2)/δY*.

We described the numbers of DCs in the skin using a phenomenological function fitted it to the data to get a descriptive curve, *X(t).* We found that the form

gave a reasonable description of the rise and fall in the numbers of DC recovered from the footpad, both in treated mice (Alum+LPS) and saline controls. We then used this curve to predict the rate at which cells enter the dLN at any given time post-challenge. At the time of photo-conversion (denoted *t_UV_*) all DCs in the footpad are assumed to be switched, *X****^∗^****(t)*, which then gradually migrate and appear as switched DC in the dLN, *Y(t)*. These cells die or leave the dLN at a combined loss rate δY.

We solved the differential equations 2 and 3 with initial conditions,

We considered scenarios in which the egress rate *μ* stays constant or changes with time post-challenge. For the latter we assumed the following functional form ,

In this model, although *μ* varies with time, DCs are still ‘memoryless’ in the sense that the skin environment dictates from moment to moment how likely DCs are to leave, irrespective of when they arrived.

**Supplemental Information 1: Further information on the mathematical modelling algorithm**
